# Impact of Column Load and Signal‐to‐Noise Threshold on the Accuracy and Repeatability of Jet A Hydrocarbon Profiling via GC×GC/FID

**DOI:** 10.1002/jssc.70356

**Published:** 2026-01-21

**Authors:** Brent A. Modereger, Louis Edwards Caceres‐Martinez, Michael E. Peretich, Hilkka I. Kenttämaa, Gozdem Kilaz

**Affiliations:** ^1^ Department of Chemistry Purdue University West Lafayette Indiana USA; ^2^ School of Engineering Technology Fuel Laboratory of Renewable Energy (FLORE) Purdue University West Lafayette Indiana USA; ^3^ Naval Air Warfare Center Aircraft Division Patuxent River Maryland USA

**Keywords:** accuracy, column load, GC×GC/FID, jet fuel, repeatability, signal‐to‐noise ratio threshold

## Abstract

Comprehensive two‐dimensional gas chromatography with flame ionization detection (GC×GC/FID) is a powerful technique for quantifying hydrocarbons in jet fuel. However, factors such as column load and the signal‐to‐noise (S/N) ratio threshold used for peak inclusion can significantly influence the accuracy and repeatability of hydrocarbon composition measurements. Despite their importance, the effects of these parameters have not been systematically studied. Accurate and precise quantitation of individual hydrocarbon groups (e.g., C_7_ isoalkanes) is particularly critical, as specific jet fuel properties, such as flash point, freezing point, and kinematic viscosity, are highly dependent on the concentrations of specific hydrocarbon classes. In this study, we investigated the influence of column load (0.05 vs. 2.5 nL) and S/N threshold (0–150) on the measured hydrocarbon composition of Jet A fuel using GC×GC/FID. Results show that both variables significantly affected the number of compounds detected, their measured weight percentages, and the repeatability of the analysis. A higher column load (2.5 nL) generally resulted in more accurate and repeatable measurements compared to a lower load (0.05 nL). In addition, an inverse relationship was found for both the accuracy and repeatability of the hydrocarbon composition measurements versus the S/N threshold value used. These findings demonstrate that GC×GC/FID measurement quality in terms of accuracy and repeatability can be optimized by maximizing the column load value (*without incurring on chromatographic performance deterioration*) and by minimizing the S/N threshold value.

## 1 Introduction

Detailed knowledge on the hydrocarbon composition of jet fuel is important as it can be used to predict many relevant chemical and physical properties of the fuel [1, 2, 3, 4]. Comprehensive two‐dimensional gas chromatography with flame ionization detection (GC×GC/FID) is a powerful analytical technique that is particularly well suited for determining the relative concentrations of hydrocarbons in mixtures because of its high peak capacity [5], the similar (± 5%) detector response of the FID for different types of hydrocarbons on a per mass basis [6], and the pattern in which different hydrocarbons in jet fuel elute from GC×GC. The elution patterns enable regions of the chromatogram to be associated with distinct hydrocarbon classes (e.g., *n*‐alkanes, isoalkanes, cycloalkanes, and aromatic compounds), which can be further classified by their number of carbon atoms, that is, hydrocarbon groups [7]. Therefore, given its widespread use in jet fuel analysis, standardizing the GC×GC/FID method is critical to ensure consistency and comparability of results [8, 9, 10, 11, 12, 13]. However, only limited knowledge exist on how the hydrocarbon compositions determined by using GC×GC/FID are affected by various experimental parameters, such as the column load value, which is the mass of jet fuel loaded onto the GC×GC columns, and the signal‐to‐noise (S/N) ratio threshold, which is the minimum S/N value a GC×GC peak must exhibit to be included for quantitation purposes. It is especially important that the concentration of individual hydrocarbon groups (isomers of the same hydrocarbon class, e.g., C_19_ isoalkanes) can be determined accurately and repeatedly because some fuel properties are highly sensitive to the concentration of compounds in specific hydrocarbon groups. In fact, it has been previously shown that jet fuel properties, such as flash point and freezing point, are primarily influenced by small hydrocarbons [6, 12] and large *n*‐alkanes [12], respectively, while large isoalkanes significantly increase the kinematic viscosity in both jet fuels and alternative blending components [4].

The column load value is determined by three GC×GC/FID method parameters: [14, 15] (1) the injection volume used for analysis, which is the volume of jet fuel sample (after dilution) injected into the GC×GC instrument; (2) the dilution factor used for sample preparation, which is the ratio of the final volume of a jet fuel sample after dilution and the volume of the jet fuel sample before dilution [16]; and (3) the split ratio used for injection (if a split injection is performed, which is an injection technique whereby only a portion of the sample injected into the inlet is transferred into the GC×GC columns and the other portion is vented from the inlet), which is the ratio of the flow rate at which the inlet is vented and the flow rate of the carrier gas through the GC×GC columns [16]. Column load values of 0.05 [6] and 0.25 nL [5, 7, 8, 10, 11] have been reported (using a S/N threshold of 25 [6], a S/N threshold of 75 [7], or an unreported S/N threshold [5, 8, 10, 11]) for determining the hydrocarbon composition of jet fuel by using GC×GC/FID. Unfortunately, sometimes column load values are not reported [3, 9, 12], which increases the ambiguity of the results and makes replicating the results difficult. In fact, the effects of the column load value on the measured hydrocarbon composition of jet fuel (or any other mixtures of hydrocarbons) have not been previously explored.

GC×GC/FID chromatograms of middle and heavy distillates often contain hundreds or thousands of peaks, which can make establishing elution boundaries for individual hydrocarbon classes or groups difficult. The S/N threshold affects the number of compounds included in quantitation and therefore can be used to simplify the GC×GC/FID chromatograms, which therefore simplifies compound identification. However, the S/N threshold also affects the accuracy and precision of the results [6]. Unfortunately, the S/N threshold values used are usually not reported in literature, with the exceptions of Vozka et al., who used S/N thresholds of 50 [2] and 75 [7], and Modereger et al., who used a S/N threshold of 25 [9]. While Modereger et al. previously examined the impact of S/N threshold on measured hydrocarbon concentrations, their study was limited to only eight S/N threshold values (0, 10, 25, 50, 75, 100, 125, 150) and a single column load (0.05 nL) [9]. The lack of knowledge concerning the S/N threshold value used increases the ambiguity of the results and makes replicating the results difficult.

In this study, the effects of the column load value and S/N threshold value on the measured weight fraction of 10 hydrocarbon classes (*n*‐alkanes, isoalkanes, monocycloalkanes, etc.) and 106 hydrocarbon groups (coverage of isomers for the different hydrocarbon classes) in Jet A were investigated. In this case, two column load values, that is, 0.05 and 2.5 nL were considered, which are equal to the lowest column load value and ten times the highest column load values reported in the literature for determining the hydrocarbon composition of jet fuel by using GC×GC/FID. In addition, 151 different S/N threshold values (from 0 to 150) were also examined. The number of GC×GC peaks and the percentage of the peak areas included in quantitation, as well as the relative standard deviation in the mass fraction (i.e., RSD wt. %) were considered for each hydrocarbon class and group in the fuel for each column load and S/N threshold value implemented.

## 2 Experimental

### 2.1 Chemicals and Jet A

Jet A fuel (POSF 10325) was provided by the United States Air Force Research Laboratory (Wright‐Patterson Air Force Base, OH). Pentane (> 98% purity) was procured from JT Baker (Radnor Township, PA). Ultra‐high purity (99.999%) helium purchased from Indiana Oxygen (Indianapolis, IN) was used as carrier gas.

### 2.2 GC×GC/FID Instrument Parameters

All GC×GC/FID measurements were performed using an instrument composed of a 7890B GC oven (Agilent, Santa Clara, CA), a 7683 series autosampler (Hewlett‐Packard, Palo Alto, CA), a 7683B series injector (Agilent, Santa Clara, CA), an FID (Agilent, Santa Clara, CA), and a quad‐jet dual‐stage thermal modulator (LECO Corporation, Saint Joseph, MI) cooled with liquid nitrogen. Reversed phase column configuration was used to improve the separation among saturated hydrocarbons [1]. The capillary column contained within the primary oven (primary column) was a midpolar 30 m × 0.25 mm × 0.25 µm DB‐17MS column (Agilent, Santa Clara, CA). The capillary column contained within the secondary oven (secondary column) was a nonpolar 0.8 m × 0.25 mm × 0.25 µm DB‐1MS column (Agilent, Santa Clara, CA). A 0.3 m guard column (Ultimate Plus Deactivated Fused Silica) was used between the secondary column and the FID. Ultra‐high purity (99.999%) helium was used as the carrier gas, with a flow rate of 1.5 mL/min. The instrument was operated using ChromaTOF software optimized for GC×GC/FID (version 4.71.0.0; LECO Corporation, Saint Joseph, MI).

### 2.3 GC×GC/FID Operating Conditions and Sample Preparation

Two different GC×GC/FID methods that were previously published were used in this study. The only major difference between these two GC×GC/FID methods was the column load value. One of the GC×GC/FID methods used a column load value of 0.05 nL [9] and is referred to as the low column load (LCL) method. The other GC×GC/FID method used a column load value of 2.5 nL [17] and is referred to as the high column load (HCL) method. All the other method parameters of the LCL and HCL methods were identical except the acquisition delay and maximum oven temperature. Therefore, the first‐ and second‐dimension retention times of each compound were the same for both methods. For both methods, primary oven temperature was maintained at 40°C for 0.2 min, increased to 185°C (LCL method) or 260°C (HCL method) at a temperature ramp rate of 3°C /min, and then held constant for 5 min. It was necessary to use a maximum primary oven temperature of 260°C for the HCL method because this method was more sensitive and therefore additional peaks were detected with first‐dimension retention times greater than 3200 s (no compounds were experimentally detected with first‐dimension retention times greater than 3200 s with the LCL method). The temperature offsets (relative to the primary oven) for the secondary oven and modulator were +50°C and +15°C, respectively. The modulation period was 2.5 s, with a hot pulse duration of 0.425 s and a cold pulse duration of 0.825 s. The temperatures of the FID and the inlet were 300°C and 280°C, respectively. The acquisition rate was 200 Hz. An acquisition delay of 130 s was used for the LCL method because Jet A was diluted 100‐fold in *n*‐pentane prior to analysis (retention time of *n*‐pentane < 130 s). No acquisition delay was used for the HCL method because Jet A was not diluted prior to analysis. The injection volume was 0.1 µL (LCL method) and 0.5 µL (HCL method). The split ratio was 20 (LCL method) or 200 (HCL method).

### 2.4 Classification of GC×GC/FID Peaks

**FIGURE 1:**
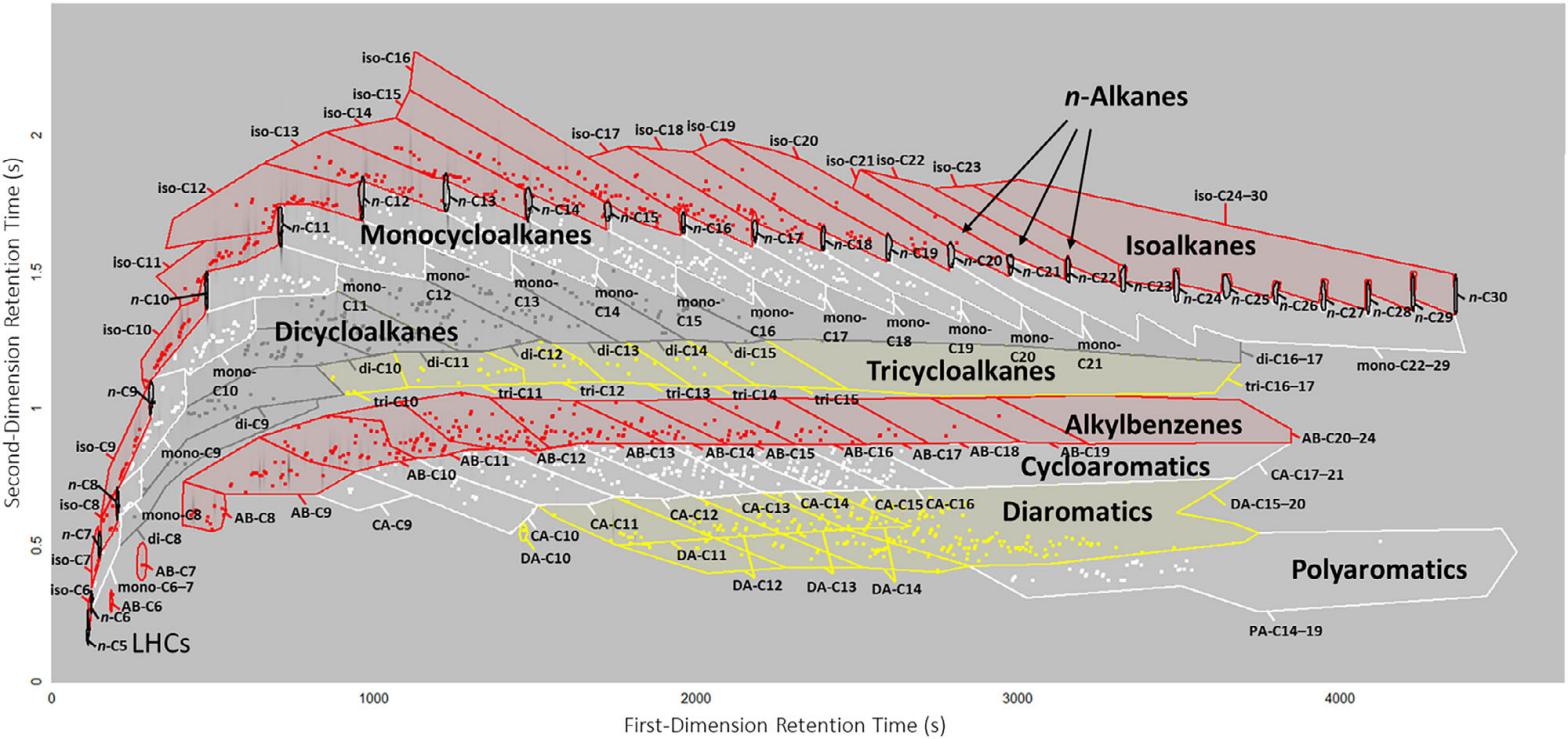
Elution boundaries for hydrocarbon classes and isomer groups. LHCs = light hydrocarbons (black and red); *n* = *n*‐alkanes (black); iso = isoalkanes (red); mono = monocycloalkanes (white); di = dicycloalkanes (grey); tri = tricycloalkanes (yellow); AB = alkylbenzenes (red); CA = cycloaromatic compounds (white); DA = diaromatic compounds (yellow); PA = polyaromatic compounds. The number after each abbreviation denotes the number of carbon atoms per molecule.

Compounds were assigned to different hydrocarbon classes and groups according to their first‐ and second‐dimension retention times, that is, according to the region of the chromatogram in which they were detected. Hydrocarbon classes (*n*‐alkanes, isoalkanes, monocyclic compounds, etc.) and groups (isomers in the same hydrocarbon class) were both investigated because their chemical and physical properties are different. All compounds were automatically grouped by the ChromaTOF software (LECO Corporation, Saint Joseph, MI) into ten hydrocarbon classes as shown in Table 1. These are light hydrocarbons (isoalkanes with six carbons and *n*‐alkanes with five or six carbons), *n*‐alkanes (with more than six carbons), isoalkanes (with more than six carbons), monocycloalkanes, dicycloalkanes, tricycloalkanes, alkylbenzenes, cycloaromatic compounds (e.g., tetralin and indane), diaromatic compounds, and triaromatic compounds, accounting for a total of 106 hydrocarbon groups. Light hydrocarbons were classified separately because they could only be analyzed with the HCL method since the first‐dimension retention times of the light hydrocarbons were lower than 130 s, which was the acquisition delay used for the LCL method. Chromatograms were separated into regions for the classification of different compound types based on elution boundaries that were previously established using hydrocarbon standards and GC×GC with positive mode electron ionization mass spectrometry (Figure 1) [9, 17]. Chromatograms were systematically examined to identify compounds that were incorrectly assigned to hydrocarbon classes or groups due to minor variations in their first‐ and/or second‐dimension retention times. These inconsistencies were corrected by making minor adjustments to the elution boundaries in such cases. No compounds were observed with first‐dimension retention times later than 3200 s when the LCL method was used; therefore, compounds with first‐dimension retention times higher than 3200 s (C_23_–C_30_ isoalkanes, C_23_–C_30_ isoalkanes, C_17_–C_19_ diaromatic compounds, and C_14_‐C_18_ polyaromatic compounds) were only quantified with the HCL method.

**TABLE 1:** The different hydrocarbon classes and groups considered for quantitation.

Hydrocarbon class	Hydrocarbon groups	# of hydrocarbon groups
Light hydrocarbons	C_5_ and C_6_ *n*‐alkanes and C6 isoalkanes as a single hydrocarbon group	1
*n*‐Alkanes	C_7_–C_30_	24
Isoalkanes	C_7–_C_30_ (C_24_–C_30_ as a single hydrocarbon group)	18
Monocycloalkanes	C_7_–C_29_ (C_22_–C_29_ as a single hydrocarbon group)	16
Dicycloalkanes	C_8_–C_17_ (C_16_ & C_17_ as a single hydrocarbon group)	9
Tricycloalkanes	C_10_–C_17_ (C_16_ & C_17_ as a single hydrocarbon group)	7
Alkylbenzenes	C_6_–C_24_ (C_20_–C_24_ as a single hydrocarbon group)	15
Cycloaromatic compounds	C_9_–C_21_ (C_17_–C_21_ as a single hydrocarbon group)	9
Diaromatic compounds	C_10_–C_20_ (C_15_–C_20_ as a single hydrocarbon group)	6
Polyaromatic compounds	C_14_–C_19_ as a single hydrocarbon group	1

### 2.5 Average Weight Fraction, RSD, GC×GC Peak Counts, and Quantitation Coverage for each Hydrocarbon Group, Class, Column Load, and S/N Threshold

**FIGURE 2:**
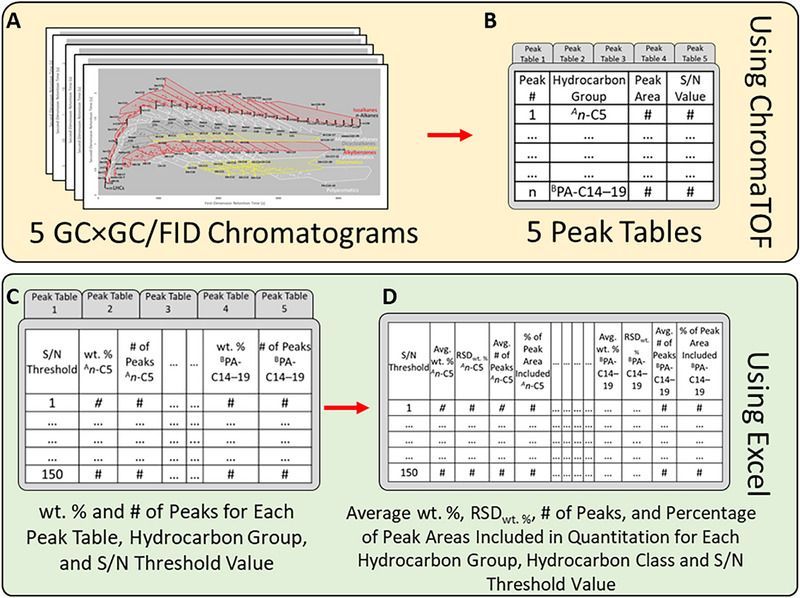
Workflow for the determination of the average wt.%, RSD wt.%, number of peaks, and percentage of peak areas included in quantitation for each hydrocarbon group, hydrocarbon class, and S/N threshold value. Workflow was used for the LCL and HCL methods separately. Abbreviations used: *n*‐C_5_ = C_5_ *n*‐alkanes; PA C_14–19_ = C_14–19_ polyaromatic compounds.

**FIGURE 3:**
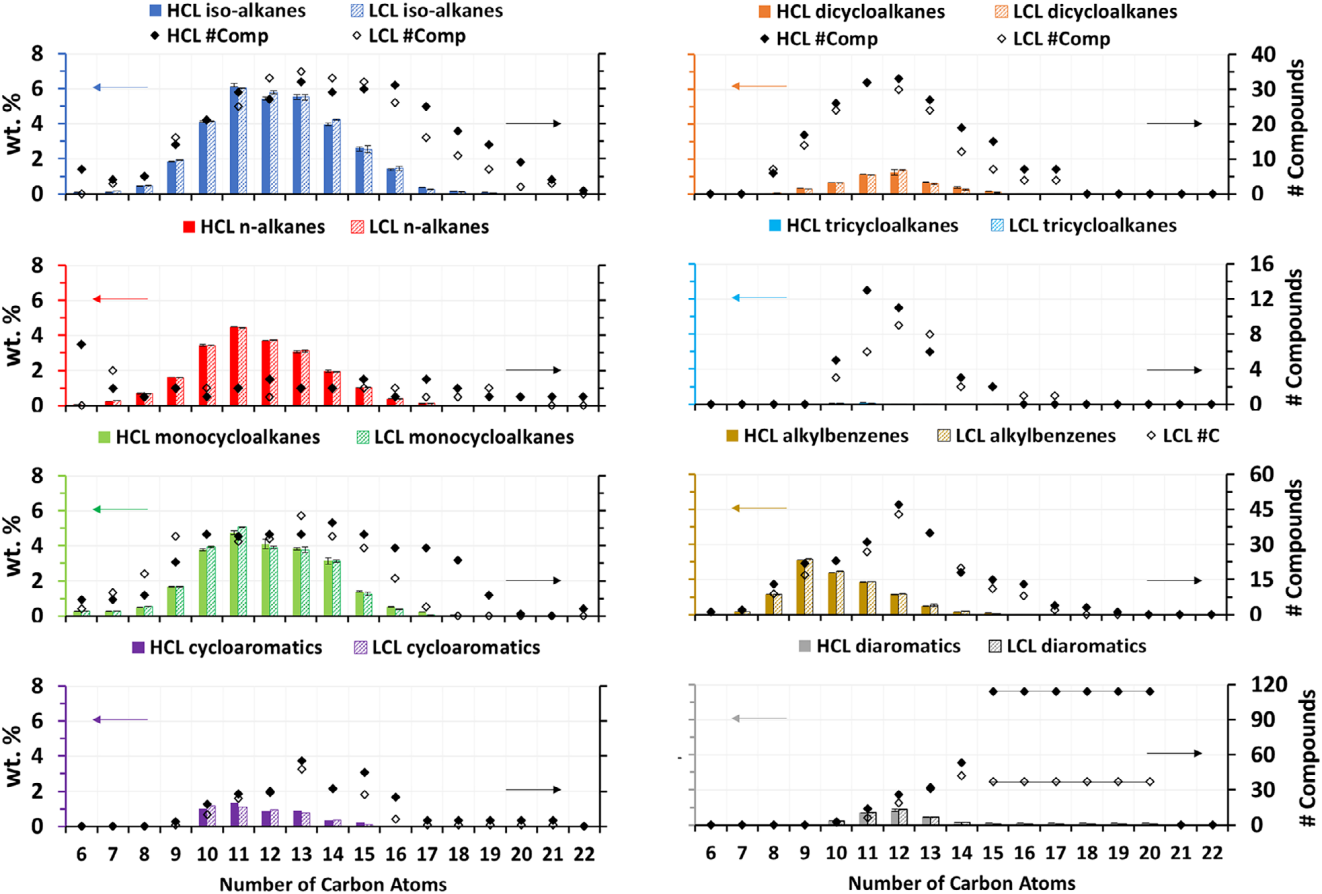
Weight fraction (wt.%) of different hydrocarbon classes and groups by using a S/N threshold value of 0. The 95% confidence intervals measured for Jet A with the HCL (colored) and LCL (pattern) methods are also shown. Markers denote the number of compounds detected for each hydrocarbon group for each loading condition.

**FIGURE 4:**
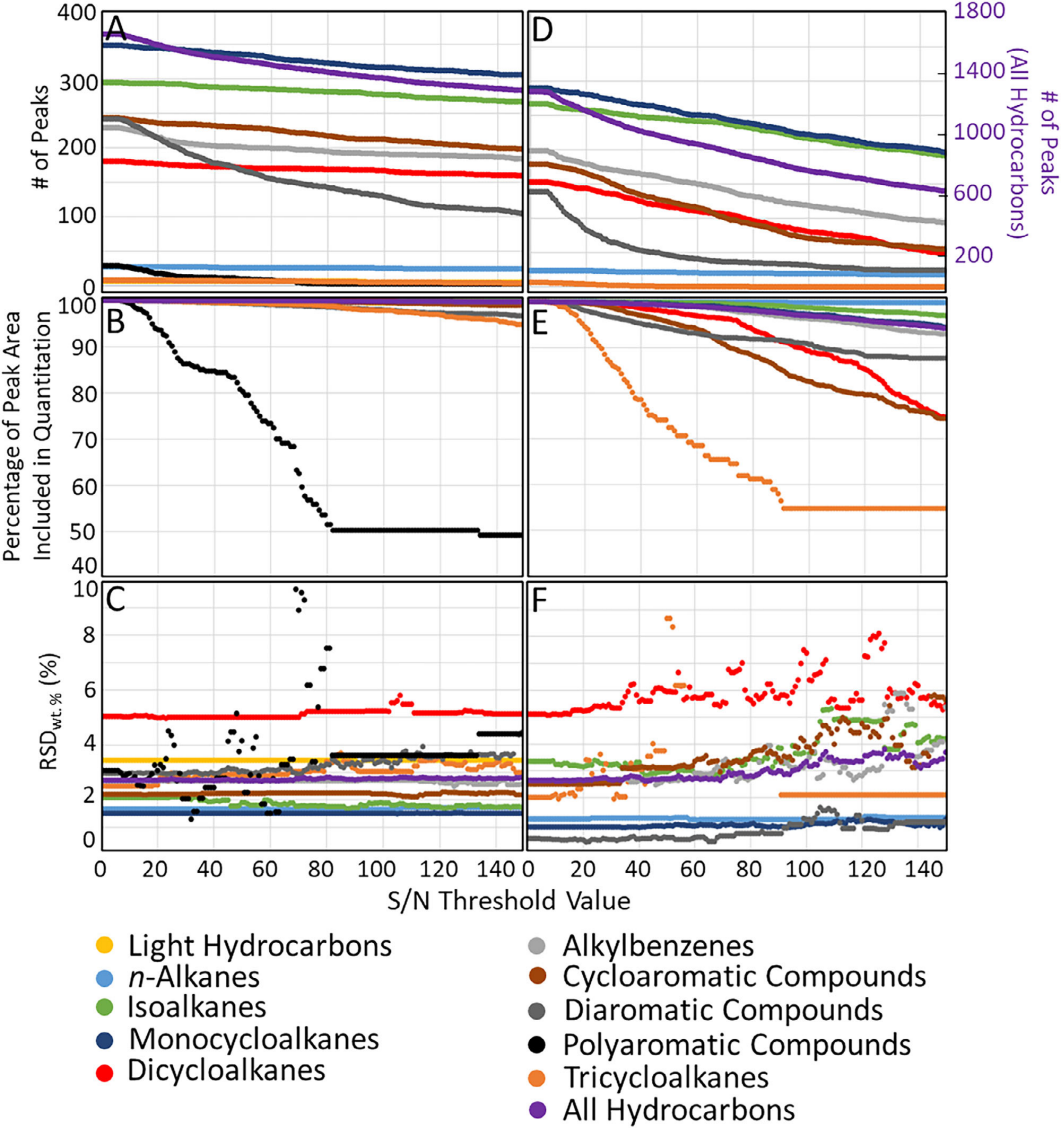
Number of compounds, percentage of peak area included in quantitation, and RSD wt.% of each hydrocarbon class when using S/N threshold values 0–150 for the HCL method (A, B, and C, respectively) and the LCL method (D, E, and F, respectively).

Jet A was analyzed five times with both the LCL and the HCL methods. ChromaTOF automatically generated a peak table for each chromatogram, which contained the area, S/N value, and hydrocarbon group for every peak (Figure 2A,B). Peak tables from each set of the five chromatograms were imported from ChromaTOF (LECO Corporation, Saint Joseph, MI) into Microsoft Excel (Microsoft, Redmond, WA). For each peak table and S/N threshold value, the number of compounds in each hydrocarbon group was determined, as well as the weight fraction (wt.%) of the compounds in each hydrocarbon group (Figure 2C). The weight fraction of the compounds was determined via peak area integration by dividing the sum of the peak areas for all the compounds in the hydrocarbon group by the total peak area of the entire chromatogram (which was possible because of the similar detector response (± 5%) of the FID for different hydrocarbons on a per mass basis) [18, 19]. From each set of five peak tables, the average number of compounds, the average wt.% of the compounds, the average percentage of peak areas included in the quantitation, and the relative standard deviation of the wt.% of the compounds were derived for each column load value, hydrocarbon group, hydrocarbon class, and S/N threshold value (Figure 2D). The average RSD wt.% was calculated for each hydrocarbon group, each hydrocarbon class, and for all the compounds together. The average number of compounds, the average percentage of the peak areas included in the quantitation, and the standard deviation of the wt.% of the compounds were plotted as a function of the S/N threshold value used for each hydrocarbon class (Figure 3) and hydrocarbon group with an average weight fraction higher than 0.01 wt.% (Figure 4 and Figures S1–S7). Although the wt.% of the C_20_ *n*‐alkanes, C_14_ tricycloalkanes, C_17_ alkylbenzenes, C_18_ alkylbenzenes, and C_17–21_ cycloaromatic compounds were each lower than 0.01 wt.%, the average number of compounds, the average percentage of the peak areas included in the quantitation, and the standard deviation of the wt.% of the compounds were also plotted (Figure 4 and Figures S1–S7) as a function of the S/N threshold used because the number of compounds and the RSD wt.% values were like those of the hydrocarbon groups with an average weight fraction higher than 0.01 wt.%.

## 3 Results and Discussion

### 3.1 Effects of the Column Load Value on the Measured Composition of each Hydrocarbon Class and Group

The column load value used for analysis was found to significantly affect the number of compounds detected. On average, 1282 compounds were detected using the LCL method and 1635 compounds were detected using the HCL method (Figure 3; S/N threshold value = 0). More compounds were detected by using the HCL method for every hydrocarbon class, especially dicycloalkanes, tricycloalkanes, cycloaromatic compounds, diaromatic compounds, and polyaromatic compounds (Figure 3). However, the additionally detected compounds had very low concentrations. The 353 compounds detected using the HCL method with the lowest wt.% and S/N values had wt.% values ≤ 0.001 and 0.002, respectively, and an average combined wt.% of 0.20 and 0.26, respectively. Therefore, the additionally detected compounds accounted for 0.20%–0.26% of the fuel by weight. Although the additionally detected compounds did not significantly affect the measured wt.% of most hydrocarbon groups, they did significantly affect the measured wt.% of 15 hydrocarbon groups (Figure 3; 18%–360% increase in wt.% determined with HCL method compared to with LCL method; C_17_–C_20_ isoalkanes; C_16_–C_18_ monocycloalkanes; C_13_–C_15_ dicycloalkanes; C_15_–C_16_ alkylbenzenes; C_15_–C_16_ cycloaromatic compounds; C_14_ and C_15_–C_20_ [combined] diaromatic compounds; and C_14_–C_18_ [combined] polyaromatic compounds). The hydrocarbon groups that were significantly affected by the column load value had especially low wt.% values and/or many more compounds were detected with the HCL method, except for the C_16_ monocycloalkanes. The wt.% of the C_16_ monocycloalkanes was not especially low, and the number of C_16_ monocycloalkanes detected was the same using the LCL and HCL methods (Figure 3; 29 compounds), which suggested that some compounds were inconsistently assigned to hydrocarbon groups. Also, slightly more (1–5 more) C_9_ isoalkanes, C_12_–C_15_ isoalkanes, C_11_ monocycloalkanes, and C_14_ monocycloalkanes were detected using the LCL method (Figure 3), which further suggests that some compounds were potentially inconsistently identified.

The column load value used for analysis also affected the number of detected hydrocarbon classes and groups. Remarkably, nine hydrocarbon groups (C_21_ and C_22_ *n*‐alkanes; C_22_ and C_24_–C_30_ isoalkanes; C_20_ and C_22_–C_29_ monocycloalkanes, C_18_ and C_19_ alkylbenzenes, and C_14_–C_19_ polyaromatic compounds) were detected using the HCL method only (Figure 3). However, the additionally detected compounds did not affect the wt.% determined for eight of these hydrocarbon groups because the combined wt.% of the additionally detected compounds was lower than 0.01 wt.% for each of the hydrocarbon groups. The additionally detected compounds did affect the wt.% determined for the C_14_–C_19_ polyaromatic compounds (which was 0.00 and 0.01 wt.% with the LCL and HCL methods, respectively) because 29 additional compounds were detected. The C_14_–C_19_ polyaromatic compounds were the only polyaromatic compounds quantified. These results, encompassing significant differences in the measured wt.% for 15 hydrocarbon groups and 353 additional compounds and 9 additional hydrocarbon groups detected, suggested that a column load value of 0.05 nL was too low to accurately determine the wt.% of all the hydrocarbon groups. Thus, these results suggest that maximizing column load, without incurring on column overloading, detector saturation, or chromatographic performance deterioration, is beneficial to determine the wt.% of all the hydrocarbon groups more accurately.

### 3.2 Effects of the S/N Threshold Value on the Measured wt.% of Different Hydrocarbon Classes and Groups

The effects of the S/N threshold value on the identified and quantified peaks and the measured wt.% of different hydrocarbon classes and groups were investigated and summarized in Figure 4 for both HCL and LCL conditions. Figure 4 evidences how the number of compounds, the wt.%, and S/N values for each compound varied significantly for each hydrocarbon group and class depending on the experimental conditions used. The average RSD wt.% for all the hydrocarbon groups (S/N threshold values 0–150) was 2.7%–2.8% for the HCL method and 2.6%–3.8% for the LCL method (Figure 4C,F). A positive correlation was generally observed between the RSD wt.% and the S/N threshold value, especially for the polyaromatic compounds (HCL method; Figure 4C), dicycloalkanes (LCL method; Figure 4F), tricycloalkanes (LCL method; Figure 4F), cycloaromatic compounds (LCL method; Figure 4F), and diaromatic compounds (LCL method; Figure 4F). This was due to a slight variation in the S/N value of each peak in different measurements, which caused some of the peaks with S/N values near the S/N threshold to be detected in only some of the replicate measurements. Thus, it is identified that applying arbitrary signal‐to‐noise (S/N) thresholds can inadvertently eliminate genuine peaks, thereby reducing reproducibility. A slightly negative correlation was observed between the RSD wt.% and the selected S/N threshold value for the isoalkanes, alkylbenzenes, and cycloaromatic compounds with the HCL method (Figure 4C), which suggested that these hydrocarbon classes contained compounds detected near an elution boundary with S/N values lower than 150 that were inconsistently identified due to minor variations in their first‐ and second‐dimension retention times. No correlation was observed between the RSD wt.% and the S/N threshold value for the light hydrocarbons, *n*‐alkanes, and monocycloalkanes measured with the HCL method (Figure 4C) and for the *n*‐alkanes measured with the LCL method (Figure 4F) because the combined wt.% of the compounds with S/N values < 150 in these hydrocarbon groups was negligible (< 0.5 wt.%).

For each hydrocarbon class, a relationship was detected between the RSD wt.% and the percentages of the peak areas included in quantitation. For each hydrocarbon class, the greatest RSD wt.% values (Figure 4C,F) corresponded to ranges of selected S/N threshold values within which the percentages of the peak areas included in quantitation varied significantly (Figure 4B,E). For example, the RSD wt.% values were particularly high (roughly three to seven times the minimum RSD wt.% value of 1.3%) for the polyaromatic compounds (only detected with the HCL method) when the S/N threshold values of 24–26, 45–55, and 69–81 were used. These ranges of S/N threshold values corresponded to particularly large variations in the percentage of the peak areas included in quantitation (up to 3.5%, 8.5%, and 16.8% difference in the peak areas included in quantitation (relative to 100%) using S/N thresholds 23–26, 44–55, and 68–81; Figure 4C). Also, for each hydrocarbon class, the lowest RSD wt.% values corresponded to ranges of S/N threshold values with minimal differences in the percentages of the peak areas included in quantitation. For example, the RSD wt.% values were particularly low (1.3–4.4 RSD wt.%) and consistent for the polyaromatic compounds when S/N threshold values of 0–10, 29–36, and 83–150 were used. These ranges of S/N threshold values corresponded to minimal differences in the percentage of the peak areas included in quantitation (0, 1%.8%, and 1.1% decrease in the peak area included in quantitation [relative to 100%] using S/N thresholds 0–10, 28–36, and 82–150; Figure 4C).

In general, the HCL method was more precise (lower RSD wt.% values) than the LCL method (Figure 4C,F) because the compounds with S/N values < 150 represented a lower percentage of the total wt.%. The wt.% of the compounds with S/N values lower than 150 were 0.000015–0.0087 when the HCL method was used, compared to 0.00016–0.14 when the LCL method was used. Also, the HCL method was generally more repeatable than the LCL method because the wt.% of 15 hydrocarbon groups (C_17_–C_20_ isoalkanes; C_16_–C_18_ monocycloalkanes; C_13_–C_15_ dicycloalkanes; C_15_–C_16_ alkylbenzenes; C_15_–C_16_ cycloaromatic compounds; C_14_ and C_15_–C_20_ [combined] diaromatic compounds; and C_14_–C_18_ [combined] polyaromatic compounds) were greater when the HCL method was used (Figure 3 and as discussed in Section 3.1). The repeatability of the LCL method was affected much more by the S/N threshold value than the repeatability of the HCL method, especially for the isoalkanes, dicycloalkanes, alkylbenzenes, and cycloaromatic compounds, which were composed of more compounds with S/N values 0–150 than the other hydrocarbon classes (Figure 4C,F). The RSD wt.% values determined in this study for each hydrocarbon class when using the LCL method were slightly greater than the RSD wt.% values that were determined in a previous study which used a slightly modified version of the LCL method (e.g., 1.7–3.2 average RSD wt.% for all the hydrocarbon groups using S/N threshold values 0, 10, 25, 50, 75, 100, 125, and 150, compared to 2.6%–3.8% average RSD wt.% for all the hydrocarbon groups using S/N threshold values 0–150 in the current study) [6]. In the previous study, elution boundaries were established after the compounds with S/N values less than the S/N threshold were excluded from quantitation (as opposed to before the compounds with S/N values less than the S/N threshold were excluded from quantitation like in the current study). This suggests that elution boundaries are easier to establish when fewer peaks are included in quantitation. Therefore, when establishing elution boundaries, the precision of the hydrocarbon composition measurements might be improved by using S/N threshold values > 0.

### 3.3 Effects of the S/N Threshold Value on the Measured Chemical Composition of Each Hydrocarbon Group

**FIGURE 5:**
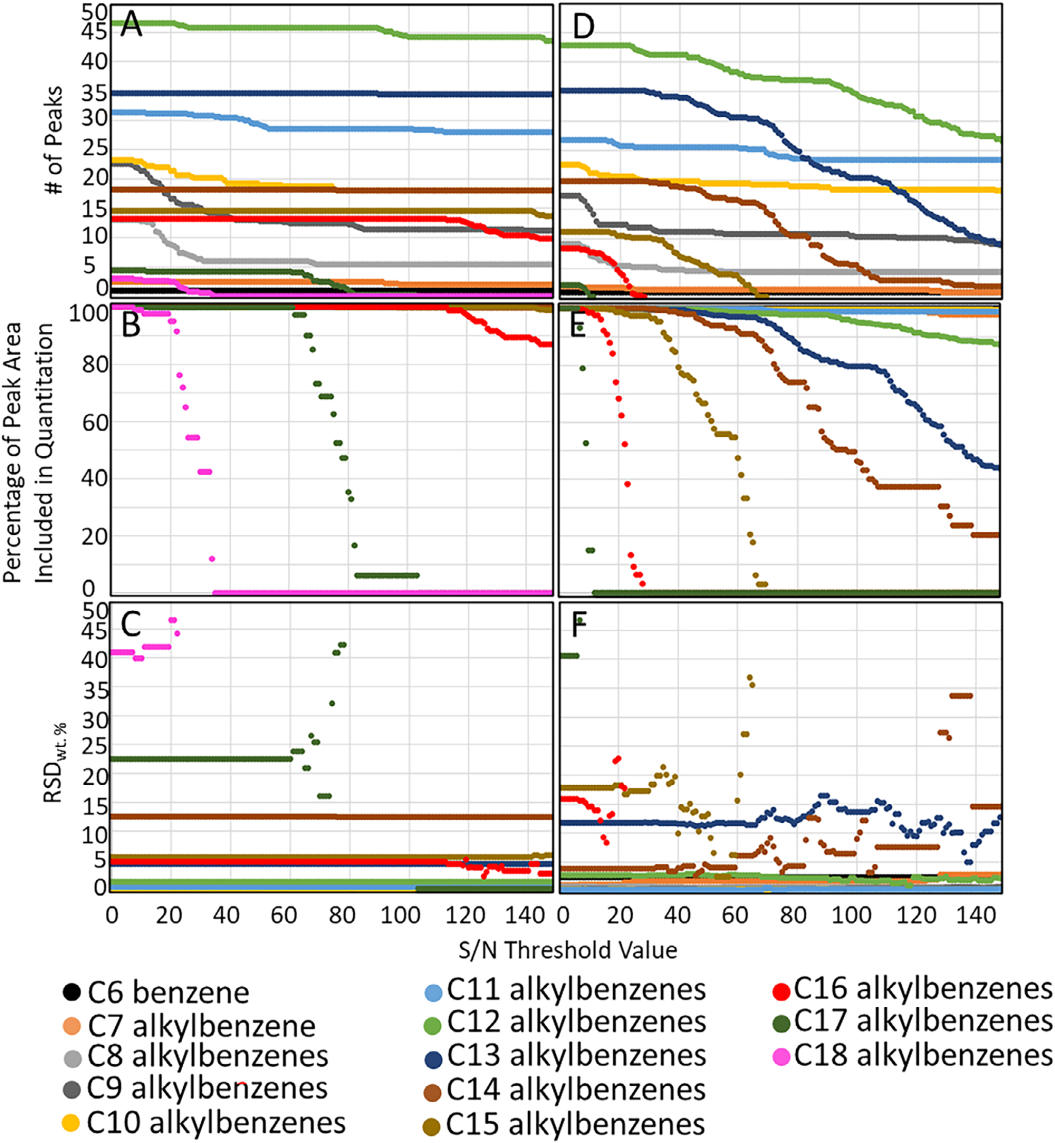
Number of compounds, percentage of peak area included for quantitation, and RSD wt.% of alkylbenzenes when using S/N threshold values 0–150 for the HCL (A, B, and C, respectively) and the LCL method (D, E, and F, respectively). The number after the name of each hydrocarbon group refers to the number of carbon atoms in each compound.

The same general trends that were detected for each hydrocarbon class were also detected for each hydrocarbon group. For example, the lower the S/N threshold used, the greater was the number of compounds included in quantitation. The same applies to the S/N threshold used and the percentage of the peak areas included in the quantitation. A positive relationship was detected between the S/N threshold used and the RSD wt.%. These relationships existed because many of the hydrocarbon groups were entirely (Table S1; 14 and 21 hydrocarbon groups when the HCL and LCL methods were used, respectively) or predominately (Table S2; three and six hydrocarbon groups when the HCL and LCL method were used, respectively) composed of compounds with S/N values < 150, especially when the LCL method was used. Also like for the hydrocarbon class measurements, the S/N threshold value affected the precision of the hydrocarbon group measurements much more for the LCL method than for the HCL method (Figures 5 and S1–S7). For example, of the hydrocarbon groups plotted in Figure 5 and Figures S1–S7 (83 hydrocarbon groups in total), an increase of ≥ 100% was observed in the RSD wt.% of 20 of the hydrocarbon groups with the LCL method, compared to six hydrocarbon groups with the HCL method (Table S3). Also, like the hydrocarbon class measurements, a relationship was observed between the percentages of the peak areas included in quantitation and the RSD wt.%. For example, for the LCL method, the RSD wt.% was especially high (roughly two to nine times higher than the minimum RSD wt.% value of 3.9%) for the C_14_ alkylbenzenes when using S/N threshold values 66–72, 83–88, 100–103, and 128–138, which corresponded to ranges of S/N threshold values within which the percentages of the peak areas included in quantitation varied significantly (up to 10.6%, 17.3%, 6.6%, and 7.0% differences in the peak areas included in quantitation (relative to 100%) using S/N thresholds 65–72, 82–88, 99–103, and 127–138; Figure 5F). Also, with the LCL method, the RSD wt.% for the C_14_ alkylbenzenes was particularly low and consistent when using S/N threshold values 0–60, 78–82, 90–99, 107–127, and 139–149, which corresponded to ranges of S/N threshold values with little or no changes in the percentages of the peak areas included in quantitation (up to 7.9%, 0.0%, 4.7%, 1.4%, and 3.3% difference in the peak area included in quantitation (relative to 100%) using S/N thresholds 0–60, 77–82, 89–99, 106–127, and 138–149; Figure 5E).

The relationship found between the wt.% included in quantitation and the RSD wt.% suggests that the precision of the measurements was negatively affected by compounds that were inconsistently included in quantitation. The effects of these inconsistencies were more pronounced for larger S/N threshold values because the compounds accounted for a greater percentage of the hydrocarbon group by wt.%. For example, with the LCL method, the highest RSD wt.% values (roughly seven to nine times the minimum RSD wt.% value of 3.9%) were found for the C_14_ alkylbenzenes with S/N threshold values 128–138 (Figure 5F). This range of S/N threshold values corresponded to the highest decrease in the percentage of the peak areas included in quantitation in relative terms (37% decrease in wt.%) but not in absolute terms (13.8 wt.%; Figure 5E). The repeatability of the measurements was also affected by the S/N threshold value used because the inherent repeatability of the measurements (which were due to normal variations in the signal response of the GC×GC/FID) increased in relation to the peak areas included in quantitation. For example, a gradual increase was observed in the RSD wt.% of the C_14_ alkylbenzenes with the LCL method when using S/N threshold values 0–60, 78–82, 107–127, and 139–149 (Figure 5F).

## 4 Conclusions

The column load value and S/N threshold value were found to significantly affect the measured hydrocarbon compositions of Jet A. The HCL method (2.5 nL) resulted in the detection of 353 more compounds and 9 more hydrocarbon groups (8 of which had wt.% values < 0.01 wt.%) than the LCL method (0.05 nL). Also, the wt.% of 15 hydrocarbon groups measured using the HCL method were found to be significantly greater than those measured using the LCL method (18%–360% increase in wt.% with HCL method compared to with LCL method). Therefore, when measuring the hydrocarbon compositions of jet fuels with GC×GC/FID, the column load value should be as large as possible (without overloading the GC×GC columns or saturating the detector) to improve the accuracy of the measurements. An inverse relationship was observed between the S/N threshold used and the RSD wt.% for each hydrocarbon class and group. Therefore, when measuring the hydrocarbon compositions of jet fuel with GC×GC/FID, the S/N threshold value should be minimized to filter out only background peaks while optimizing the detection coverage of all hydrocarbon classes (Figure 5).

## Author Contributions

**Brent A Modereger**: project administration, conceptualization, methodology, data collection, data analysis, writing – original draft preparation, reviewing and editing. **Louis Edwards Caceres‐Martinez**: data analysis, writing – reviewing and editing. **Michael E. Peretich**: technical guidance, funding acquisition. **Hilkka I. Kenttämaa**: supervision, funding acquisition, writing – reviewing and editing. **Gozdem Kilaz**: supervision, funding acquisition, writing – reviewing and editing.

## Funding

This research was made possible with the funding received by the Office of Naval Research under the award number of N000142012222.

## Conflicts of Interest

The authors declare no conflicts of interest.

## Supporting information




**Supporting File**: jssc70356‐sup‐0001‐SuppMat.docx.
