# Supplementary material for: Impact of Column Load and Signal‐to‐Noise Threshold on the Accuracy and Repeatability of Jet A Hydrocarbon Profiling via GC×GC/FID
Source: J Sep Sci. 2026 Jan 21;49(1):e70356. doi: 10.1002/jssc.70356 (PMC12824433; doi:10.1002/jssc.70356)
Supplement: Supplementary file 1 — Supporting File: jssc70356‐sup‐0001‐SuppMat.docx. [file JSSC-49-e70356-s001.docx]

**SUPPLEMENTARY INFORMATION**

**Impact of Column Load and Signal-to-Noise Threshold on the Accuracy and Repeatability of Jet A Hydrocarbon Profiling via GC×GC/FID**

Brent A. Modereger,^a^ Louis Edwards Caceres-Martinez,^b^ Michael E. Peretich,^c^ Hilkka I. Kenttämaa,^a^ Gozdem Kilaz^c*^

^a^ Department of Chemistry, Purdue University, West Lafayette 47907, IN, USA

^b^ School of Engineering Technology, Fuel Laboratory of Renewable Energy (FLORE), Purdue University, West Lafayette 47907, IN, USA

^c^ Naval Air Warfare Center Aircraft Division, Patuxent River 20670, MD, USA

* Corresponding Author: [gkilaz@purdue.edu](mailto:gkilaz@purdue.edu)

Table S1. Hydrocarbon groups that especially influenced the relationship between the S/N threshold used and the RSD_wt. %_ because they were composed entirely of compounds with S/N values < 150. The number after the name of each hydrocarbon group refers to the number of carbon atoms per compound.

| **HCL method^A^** | | | | **LCL method^B^** | | |
| --- | --- | --- | --- | --- | --- | --- |
| **Hydrocarbon group** | **wt. % of Hydrocarbon group > 0.01 wt. %^C^** | **Plotted in Figure 4 and S1–S7**  **(Yes or No)** | **Detected with LCL method**  **(Yes or No)** | **Hydrocarbon**  **group** | **wt. % of Hydrocarbon group > 0.01 wt. %^C^** | **Plotted in Figure 4 and S1–S7**  **(yes or no)** |
| *n*-C21^D^ | No | No | No | *n*-C19^D^ | Yes | Yes |
| *n*-C22^D^ | No | No | No | *n*-C20^D^ | Yes | Yes |
| iso-C21^E^ | No | No | No | iso-C20^E^ | Yes | Yes |
| iso-C22^E^ | No | No | No | monocyclo-C16^F^ | Yes | Yes |
| monocyclo-C19^F^ | No | No | No | monocyclo-C17^F^ | Yes | Yes |
| monocyclo-C20^F^ | No | No | No | monocyclo-C18^F^ | Yes | Yes |
| monocyclo-C22–26^F^ | No | No | No | dicyclo-C16–17^G^ | No | No |
| dicyclo-C16-17^G^ | No | No | Yes | tricyclo-C12^H^ | Yes | Yes |
| tricyclo-C15^H^ | No | No | Yes | tricyclo-C13^H^ | Yes | Yes |
| tricyclo-C16–17^H^ | No | No | Yes | tricyclo-C14^H^ | No | No |
| alkylbenzenes-C17 | Yes | Yes | Yes | tricyclo-C15^H^ | No | No |
| alkylbenzenes-C18 | Yes | Yes | No | tricyclo-C16-17^H^ | No | No |
| alkylbenzenes-C19 | Yes | No | No | alkylbenzenes-C15 | Yes | Yes |
| cycloaro-C17–21^I^ | No | Yes | Yes | alkylbenzenes-C16 | Yes | Yes |
| - |  | - |  | alkylbenzenes-C17 | No | Yes |
| - |  | - |  | cycloaro-14^I^ | Yes | Yes |
| - |  | - |  | cycloaro-15^I^ | Yes | Yes |
| - |  | - |  | cycloaro-16^I^ | Yes | Yes |
| - |  | - |  | cycloaro-17–21^I^ | No | No |
| - |  | - |  | diaro-C14^J^ | Yes | Yes |
| - |  | - |  | diaro-C15–20^J^ | Yes | Yes |

Acronyms used: ^A^HCL = high column load; ^B^LCL = low column load; ^C^wt. % = weight percentage; ^D^*n* = *n*-alkanes; ^E^iso = isoalkanes; ^F^monocyclo = monocycloalkanes; ^G^dicyclo = dicycloalkanes; ^H^tricyclo = tricycloalkanes; ^I^cycloaro = cycloaromatic compounds; ^J^diaro = diaromatic compounds.

Table S2. Hydrocarbon groups that especially influenced the relationship between the S/N threshold used and the RSD_wt. %_ because they were composed predominately (> 50 %) of compounds with S/N values < 150. The number after the name of each hydrocarbon group refers to the number of carbons atoms per compound.

| **HCL method^A^** | | | **LCL method^B^** | | |
| --- | --- | --- | --- | --- | --- |
| **Hydrocarbon Group** | **wt. % of Compounds with**  **S/N Values < 150^C^** | **Hydrocarbon Group** | | **wt. % of Compounds with**  **S/N Values < 150^C^** |  |
| cycloaro-C16^D^ | 98 % | iso-C17^G^ | | 86 % |  |
| diaroc-C14–C18^E^ | 55 % | dicylo-C14^H^ | | 60 % |  |
| Polyaro-C14–C18^F^ | 51 % | dicylo-C15^H^ | | 70 % |  |
| - | - | alkylbenzenes-C13 | | 56 % |  |
| - | - | alkylbenzene-C14 | | 82 % |  |
| - | - | cycloaro-C13^D^ | | 66 % |  |

Acronyms used: ^A^HCL = high column load; ^B^LCL = low column load; ^C^wt. % = weight percentage; ^D^cycloaro = cycloaromatic compounds; ^E^diaro = diaromatic compounds; ^F^polyaro = polyaromatic compounds; ^G^iso = isoalkanes; ^H^dicyclo = dicycloalkanes.

Table S3. Hydrocarbon groups with ≥ 100 % Increase in RSDwt. %. The number after the name of each hydrocarbon group refers to the number of carbons atoms per compound.

| **HCL method^A^** | | | **LCL method^B^** | |
| --- | --- | --- | --- | --- |
| **Hydrocarbon group** | **wt. % of Hydrocarbon group > 0.01 wt. %^C^** | **Detected with LCL method**  **(yes or no)** | **Hydrocarbon group** | **wt. % of Hydrocarbon group > 0.01 wt. %^C^** |
| monocyclo-C18^D^ | Yes | Yes | *n*-C19^H^ | Yes |
| tricyclo-C12^E^ | Yes | Yes | *n*-C20^H^ | No |
| alkylbenzenes-C17 | No | Yes | iso-C17^I^ | Yes |
| alkylbenzenes-C18 | No | No | monocyclo-C15^D^ | Yes |
| cycloaro-C17–21^F^ | No | Yes | monocyclo-C16^D^ | Yes |
| diaro-C15–20^G^ | Yes | Yes | monocyclo-C17^D^ | Yes |
| - | - |  | dicylo-C16–17^J^ | Yes |
| - | - |  | tricyclo-C12^E^ | Yes |
| - | - |  | tricyclo-C13^E^ | Yes |
| - | - |  | alkylbenzenes-C14 | Yes |
| - | - |  | alkylbenzenes-C15 | Yes |
| - | - |  | alkylbenzenes-C16 | Yes |
| - | - |  | alkylbenzenes-C17 | No |
| - | - |  | cycloaro-C13^F^ | Yes |
| - | - |  | cycloaro-C14^F^ | Yes |
| - | - |  | cycloaro-C15^F^ | Yes |
| - | - |  | cycloaro-C16^F^ | Yes |
| - | - |  | diaro-C13^G^ | Yes |
| - | - |  | diaro-C14^G^ | Yes |
| - | - |  | diaro-C15–20^G^ | Yes |

Acronyms used: ^A^HCL = high column load; ^B^LCL = low column load; ^C^wt. % = weight percentage; ^D^monocyclo = monocycloalkanes; ^E^tricyclo = tricycloalkanes; ^F^cycloaro = cycloaromatic compounds; ^G^diaro = diaromatic compounds; ^H^*n* = *n*-alkanes; ^I^iso = isoalkanes; ^J^dicyclo = dicycloalkanes.


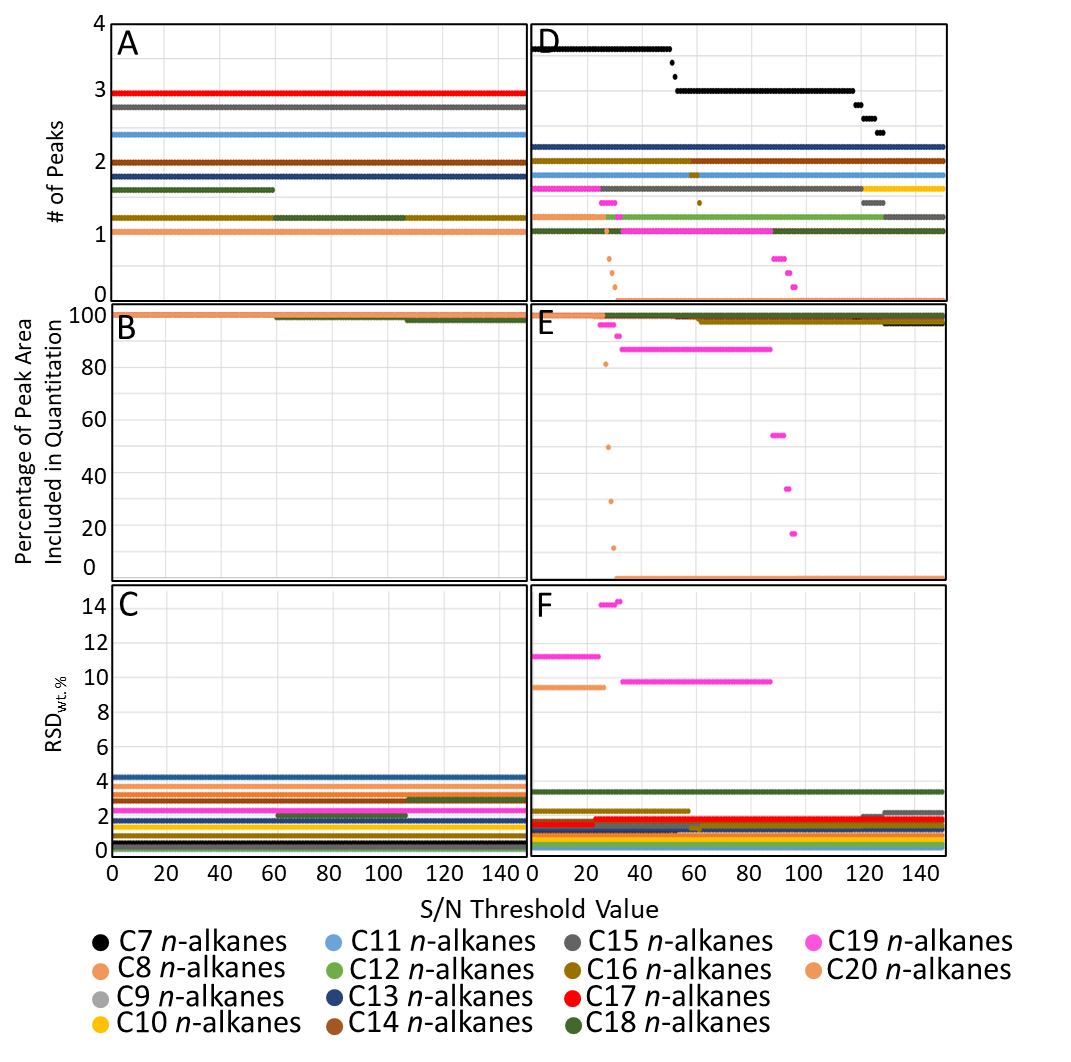
 Figure S1. Number of compounds, percentage of peak areas included in quantitation, and RSD_wt. %_ of the *n*-alkanes when using S/N threshold values 0–150 for the HCL (A, B, and C, respectively) and LCL (D, E, and F, respectively) methods. The number after the name of each hydrocarbon group refers to the number of carbon atoms in each compound. Abbreviation used: *n* = *n*-alkanes.


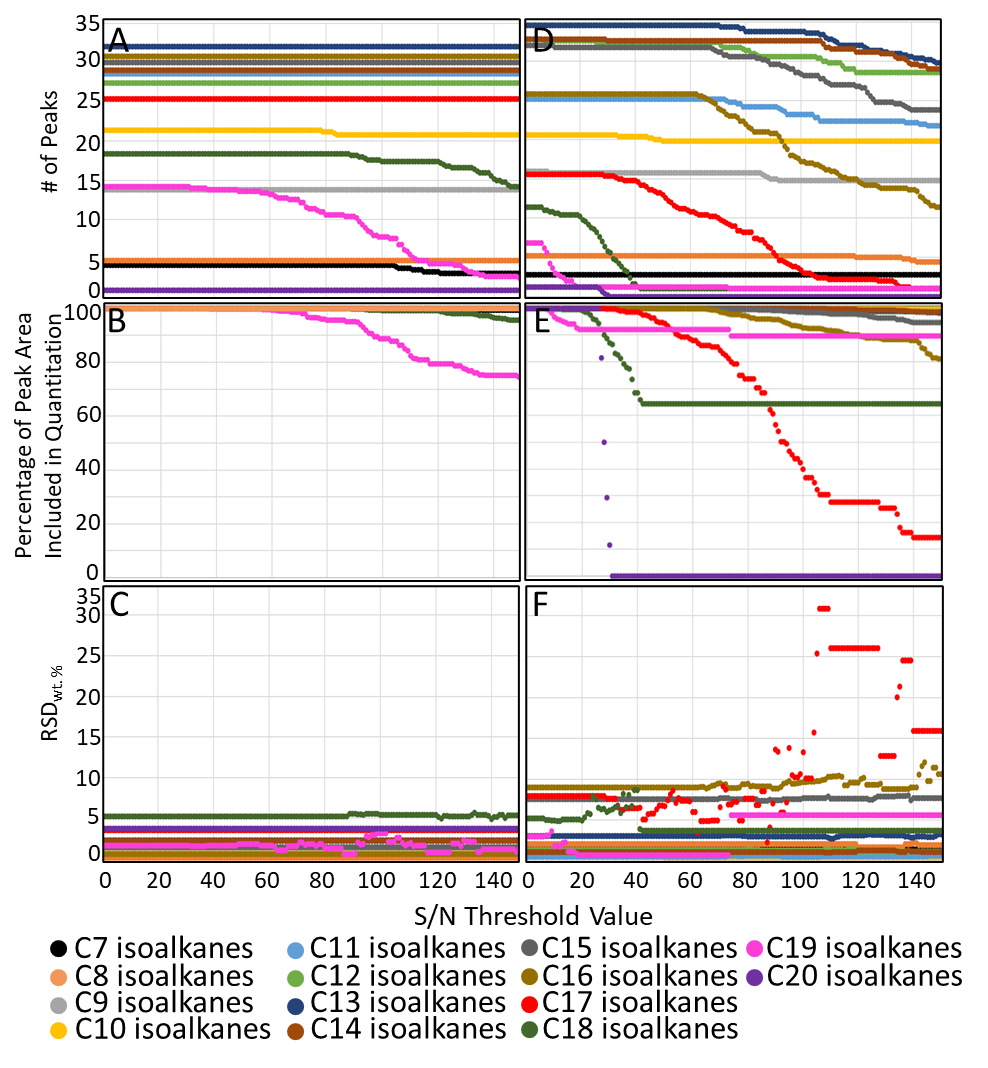
 Figure S2. Number of compounds, percentage of peak areas included for quantitation, and RSD_wt. %_ of the isoalkanes when using S/N threshold values 0–150 for the HCL (A, B, and C, respectively) and LCL (D, E, and F, respectively) methods. The number after the name of each hydrocarbon group refers to the number of carbon atoms in each compound. Abbreviation used: iso = isoalkanes.


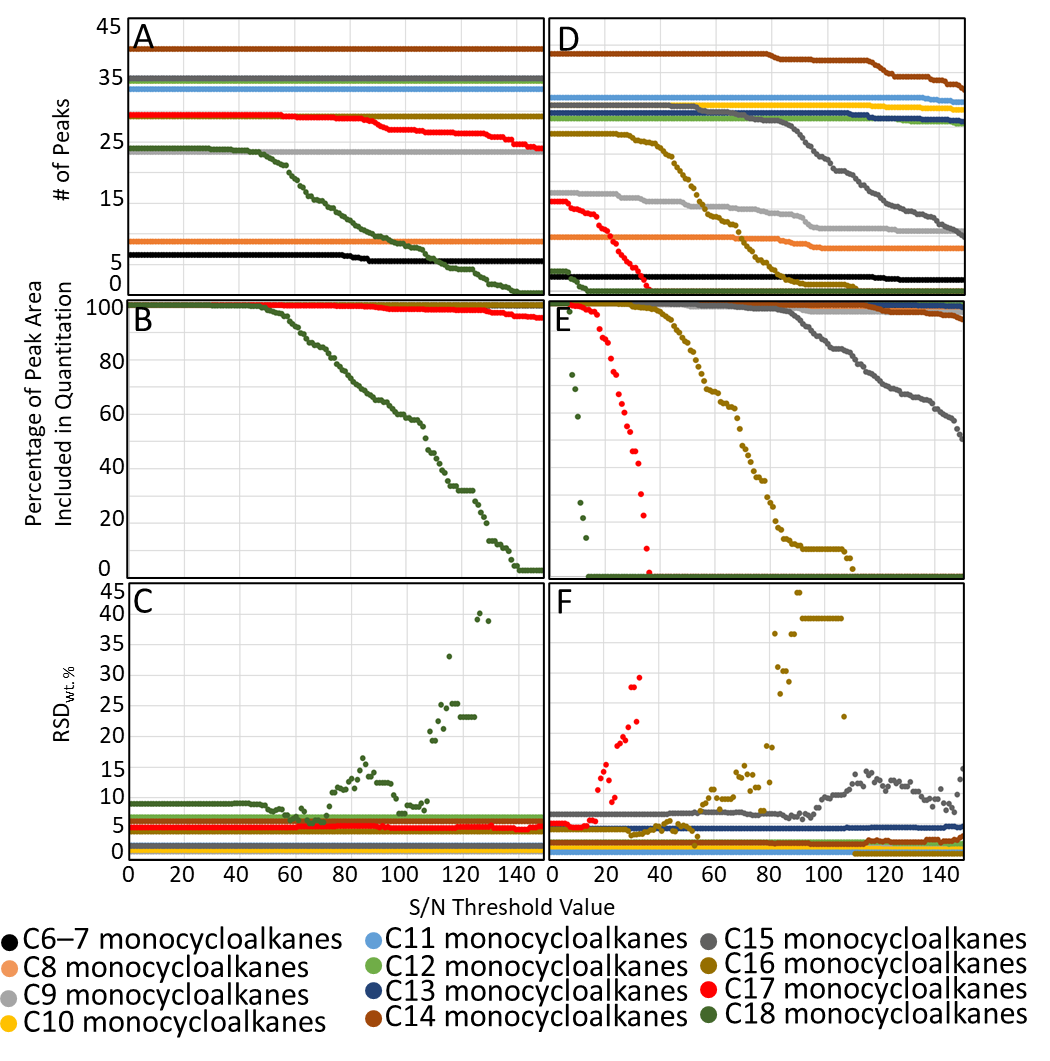
 Figure S3. Number of compounds, percentage of peak areas included for quantitation, and RSD_wt. %_ of the monocycloalkanes when using S/N threshold values 0–150 for the HCL (A, B, and C, respectively) and LCL (D, E, and F, respectively) methods. The number after the name of each hydrocarbon group refers to the number of carbon atoms in each compound. Abbreviation used: monocyclo = monocycloalkanes.


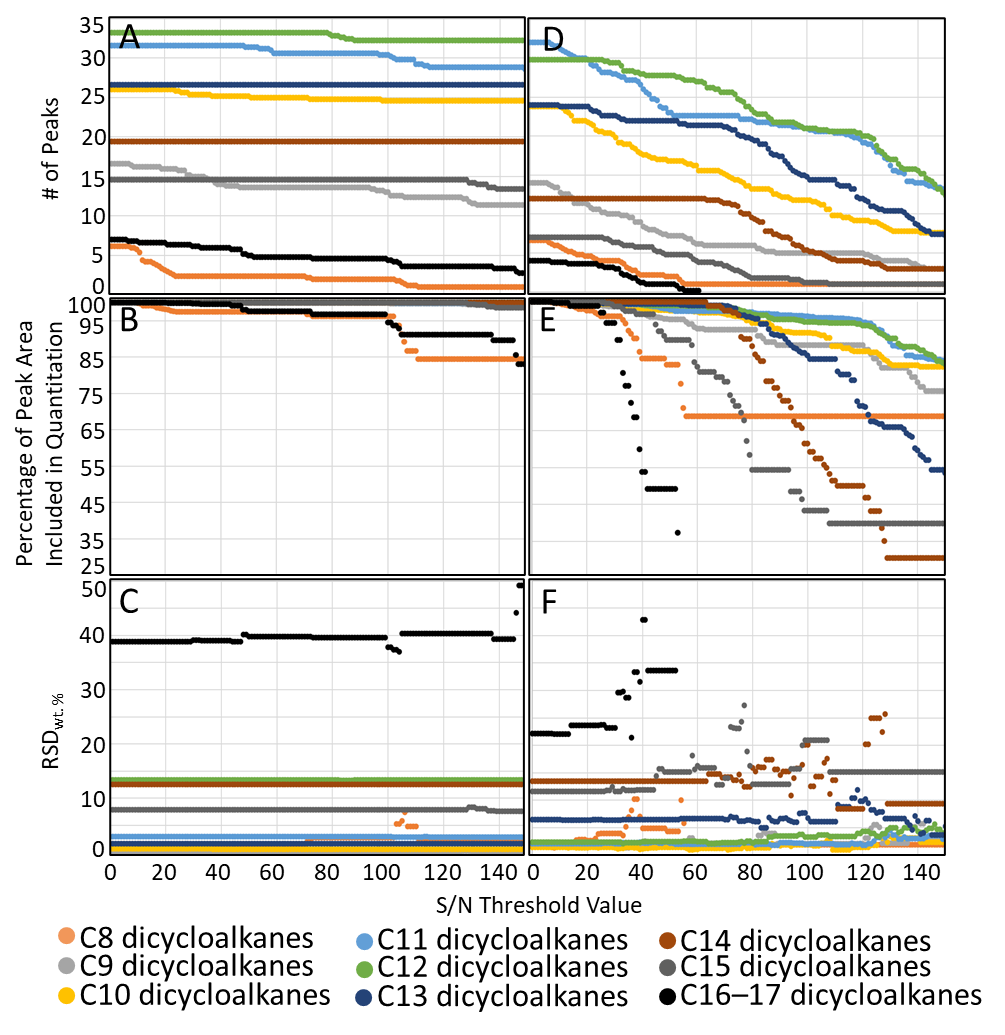
 Figure S4. Number of compounds, percentage of peak areas included for quantitation, and RSD_wt. %_ of the dicycloalkanes when using S/N threshold values 0–150 for the HCL (A, B, and C, respectively) and LCL (D, E, and F, respectively) methods. The number after the name of each hydrocarbon group refers to the number of carbon atoms in each compound. Abbreviation used: dicyclo = dicycloalkanes.


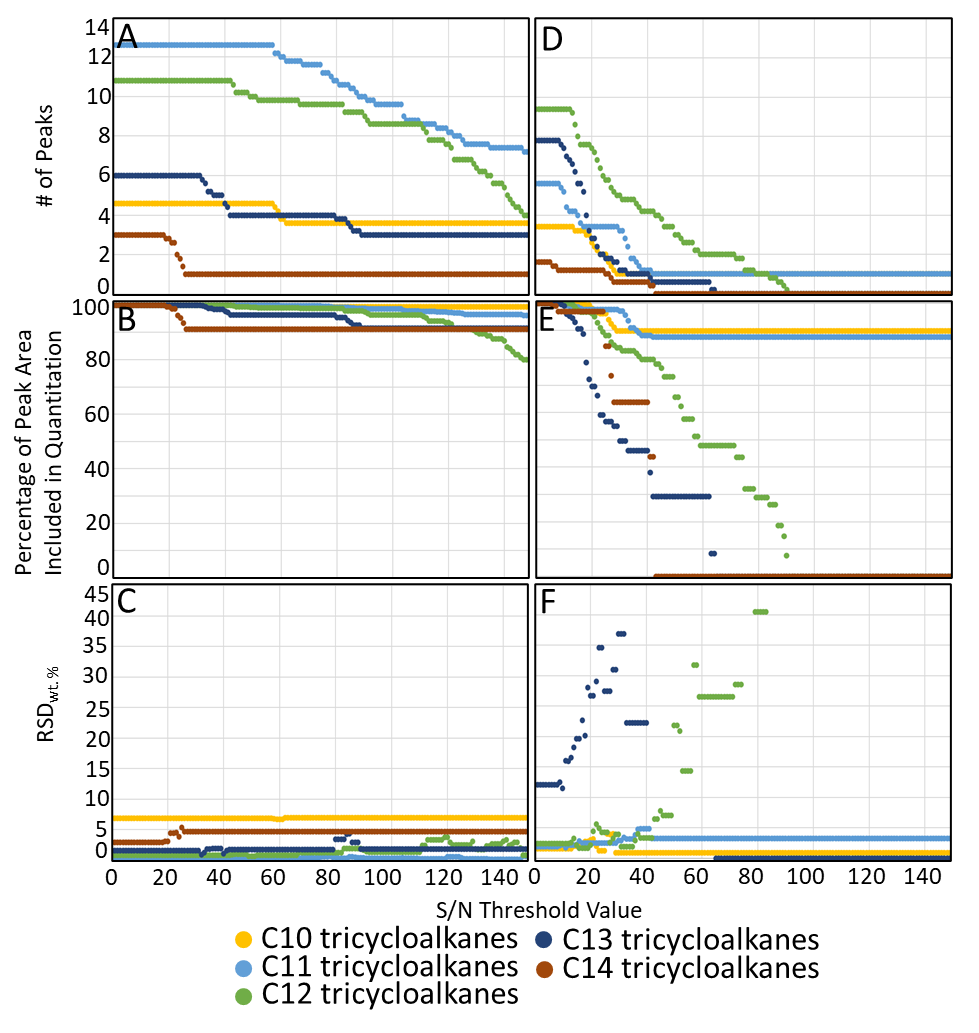
 Figure S5. Number of compounds, percentage of peak areas included for quantitation, and RSD_wt. %_ of the tricycloalkanes when using S/N threshold values 0–150 for the HCL (A, B, and C, respectively) and LCL (D, E, and F, respectively) methods. The number after the name of each hydrocarbon group refers to the number of carbon atoms in each compound. Abbreviation used: tricyclo = tricycloalkanes.


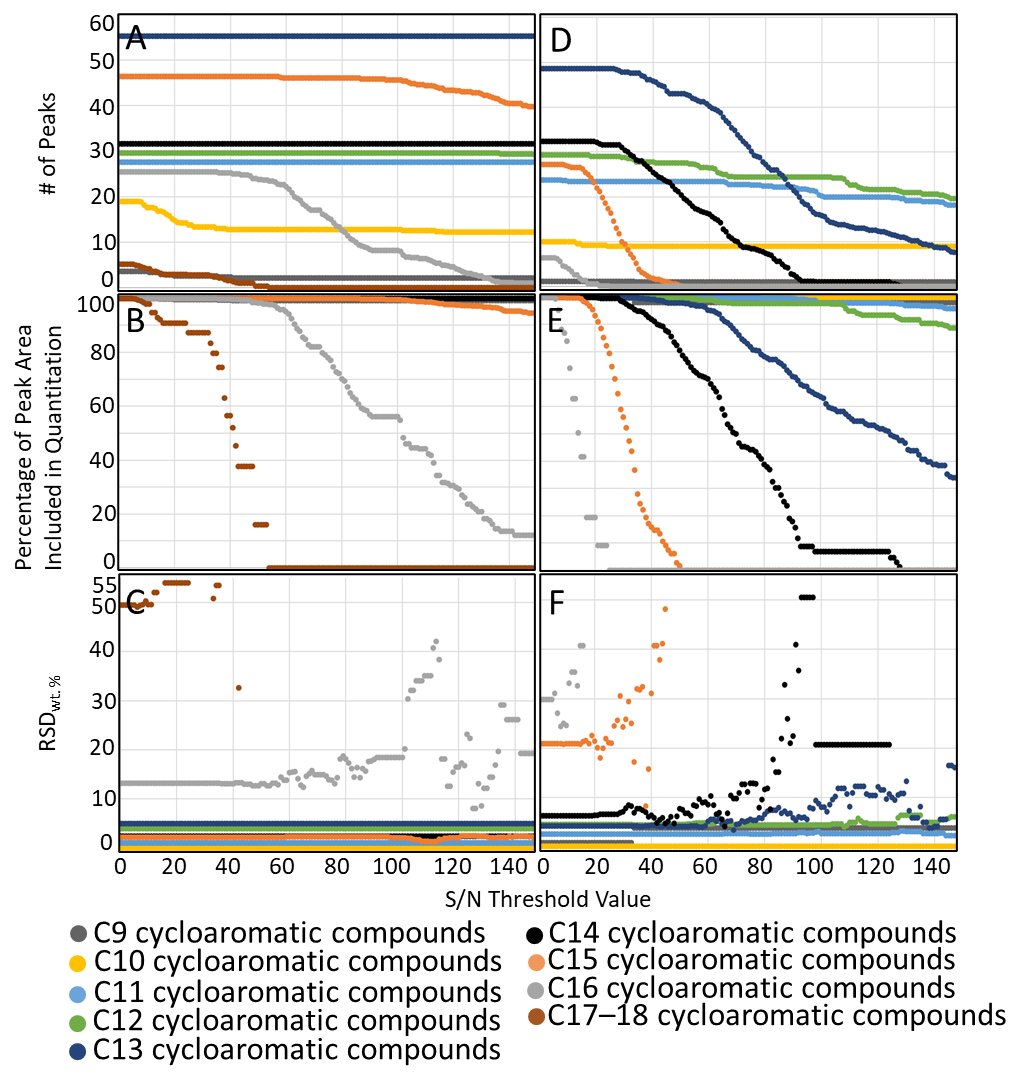
 Figure S6. Number of compounds, percentage of peak areas included for quantitation, and RSD_wt. %_ of the cycloaromatic compounds when using S/N threshold values 0–150 for the HCL (A, B, and C, respectively) and LCL (D, E, and F, respectively) methods. The number after the name of each hydrocarbon group refers to the number of carbon atoms in each compound. Abbreviation used: cycloaro = cycloaromatic compounds.


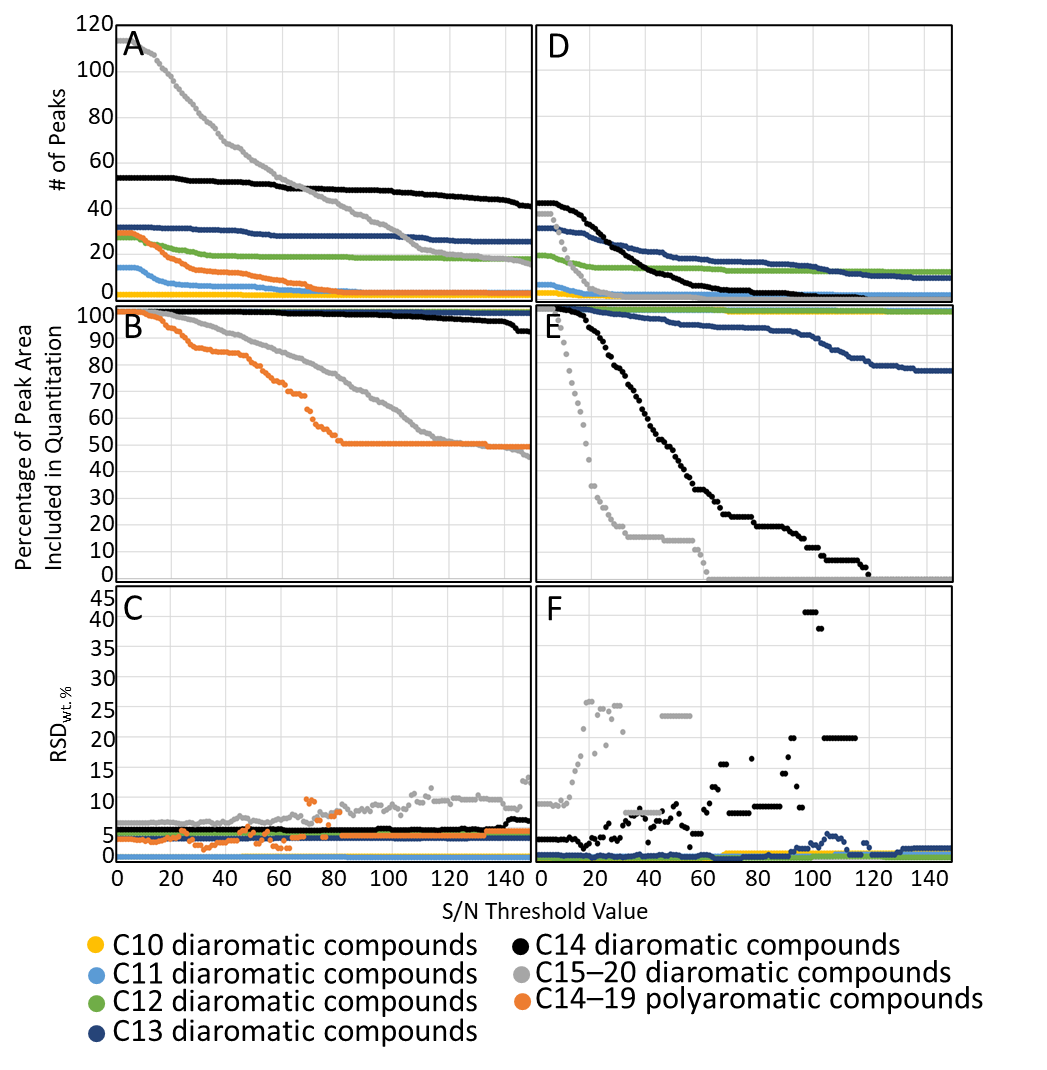
 Figure S7. Number of compounds, percentage of peak areas included for quantitation, and RSD_wt. %_ of the di- and tri/tetra-aromatic compounds when using S/N threshold values 0–150 for the HCL (A, B, and C, respectively) and LCL (D, E, and F, respectively) methods. The number after the name of each hydrocarbon group refers to the number of carbon atoms in each compound. Abbreviations used: diaromatic = diaromatic compounds; polyaromatic = polyaromatic compounds.
